# Alienness: Rapid Detection of Candidate Horizontal Gene Transfers across the Tree of Life

**DOI:** 10.3390/genes8100248

**Published:** 2017-09-29

**Authors:** Corinne Rancurel, Ludovic Legrand, Etienne G. J. Danchin

**Affiliations:** 1 INRA, CNRS, ISA, Université Côte d’Azur, 06903 Sophia Antipolis Cedex, France; corinne.rancurel@inra.fr; 2 LIPM, INRA, CNRS, Université de Toulouse, 31326 Castanet-Tolosan Cedex, France; ludovic.legrand@inra.fr

**Keywords:** horizontal gene transfer, alien index, lateral gene transfer

## Abstract

Horizontal gene transfer (HGT) is the transmission of genes between organisms by other means than parental to offspring inheritance. While it is prevalent in prokaryotes, HGT is less frequent in eukaryotes and particularly in Metazoa. Here, we propose Alienness, a taxonomy-aware web application available at http://alienness.sophia.inra.fr. Alienness parses BLAST results against public libraries to rapidly identify candidate HGT in any genome of interest. Alienness takes as input the result of a BLAST of a whole proteome of interest against any National Center for Biotechnology Information (NCBI) protein library. The user defines recipient (e.g., Metazoa) and donor (e.g., bacteria, fungi) branches of interest in the NCBI taxonomy. Based on the best BLAST E-values of candidate donor and recipient taxa, Alienness calculates an Alien Index (AI) for each query protein. An AI > 0 indicates a better hit to candidate donor than recipient taxa and a possible HGT. Higher AI represent higher gap of E-values between candidate donor and recipient and a more likely HGT. We confirmed the accuracy of Alienness on phylogenetically confirmed HGT of non-metazoan origin in plant-parasitic nematodes. Alienness scans whole proteomes to rapidly identify possible HGT in any species of interest and thus fosters exploration of HGT more easily and largely across the tree of life.

## 1. Introduction

Horizontal gene transfer (HGT), is the transmission of genes between organisms by other way than direct (vertical) inheritance from parental lineages to their offspring. HGT is prevalent in prokaryotes [1], with substantial proportions of bacterial genes jumping horizontally, rather than being vertically inherited [2]. These horizontally-acquired genes play important functions in bacteria, including spreading of antibiotic resistance and emergence of pathogenicity [3,4,5,6]. Although HGT is much less prevalent in eukaryotes, and particularly multicellular eukaryotes, there are reported cases in the literature, including in viridiplantae and Metazoa [7,8,9,10,11,12]. Some of the reported examples also evoke important associated roles in the recipient organism. This suggests that the genomic and biological impact of HGT could be more widespread in the tree of life than initially thought [13].

These HGT challenge the view of a purely tree-like backbone underlying inheritance of genetic information across species [14]. With the technological progress and cost reduction for genome sequencing, more and more genomes from a wider diversity of organisms now become available and this trend will continue. To provide a more comprehensive view of the contribution of HGT to the genomes of the different organisms across the tree of life, methods to rapidly detect candidate HGT are needed.

Two classical ways to identify candidate HGT are (i) to study the intrinsic (e.g., GC content, codon usage distribution) and/or (ii) the extrinsic (e.g., percent identity, BLAST [15] E-value against other species) characteristics of the genes of a species of interest. For instance, in the ‘intrinsic’ category, genes that have GC content and codon usage deviating from the rest of the genes of a species of interest (receiver) might be considered as a sign for horizontal acquisition. Similarly, in the ‘extrinsic’ category, if a gene from a species of interest (receiver) shows higher similarity (lower E-value, higher bit-score in BLAST) to sequences from distantly related (donor) species than to genes of close relatives; this gene is a candidate HGT. Another frequent ‘extrinsic’ approach is to assess the phylogenomic distribution of genes across a panel of diverse organism by clustering genes in groups of orthologs and looking for those with a patchy distribution (phylogenomic profiles). For a more comprehensive overview of the different categories of methods to detect HGT, please refer to this recent review by Ravenhall et al. [16].

Methods in the intrinsic category can be efficient to identify HGT, as long as the GC content and codon usage distributions of the receiver and donor genomes are sufficiently different. However, if these intrinsic metrics are closely related or if the HGT event is ancient and the acquired gene has adapted to the GC content and codon usage of the receiver organism, such methods will not be able to identify HGT [17].

Methods in the extrinsic category do not suffer from undistinguishable GC content and codon usage as they rely only in a difference of magnitude in the BLAST or other similarity metrics between closely related and distant taxa. These methods are better suited to identify HGT between distant species (e.g., trans-kingdom) and require an as comprehensive as possible reference sequence library covering a diversity of species. One such method in the extrinsic category is the Alien Index metrics. It was first introduced by Gladyshev et al. [9] to identify HGT of non-metazoan origin in a metazoan species, the bdelloid rotifer *Adineta vaga*. Briefly, an Alien Index (AI) is calculated to measure a difference of magnitude between the best non-metazoan and best metazoan E-value. If the best non-metazoan E-value is closer to 0 than the best metazoan E-value, the AI will be positive, if the AI is ≥45, it has been assumed that an HGT event is very likely. This AI method was used again to assess the total contribution of genes of non-metazoan origin in the whole genome of the bdelloid rotifer once it was sequenced [18]. Using custom Perl scripts, our laboratory and collaborators also used AI to identify candidate HGT of non-metazoan origin in the genome of the plant-parasitic nematode *Globodera rostochiensis* [19], in the genomes of several panagrolaimid nematodes [20] as well as in the transcriptomes of the nematodes *Nacobbus aberrans* [21], *Xiphinema index* [22] and other as yet unpublished genomes. Calculation of AI scores for whole proteomes had previously been implemented in a software called AlienG [23]. It was used to highlight the importance of HGT in the colonization of land by plants [8] and in several other studies of HGT across different lineages [24,25,26]. In the original AlienG, publication, an AI > 30 was deemed good indicator of acquisition via HGT. However, as far as we know, this software is neither publicly available for download nor deployed on a web server. Hence, no user-friendly web tool or downloadable software is available, so far to compute AI scores directly from BLAST results. Furthermore, extracting the taxonomic information from BLAST results against the National Center for Biotechnology Information´s (NCBI) (Bethesda, MD, USA) protein libraries can be a long and difficult task, which prevents popularization of such methods.

To circumvent this difficulty of retrieving taxonomic information, it is tempting to divide the NCBI’s non-redundant protein library (NR) or other sequence libraries into taxonomic subsets (i.e., a non-alien subset consisting of sequences from species closely related to the receiver species of interest, and one or several subsets consisting of sequences from distantly related (alien) candidate donors). This approach has been implemented in a Perl software named alien_index [27]. However, E-values obtained from BLAST against different sequence libraries are not comparable which makes calculation of an AI questionable, especially if the libraries are of different sizes. This is one of the reasons why, another score, named HGT index (or *h*), has been proposed [28]. The principle is very similar to the AI score, except that it is calculated based on a difference between the best donor and recipient species bit scores. In contrast to E-values, bit scores are comparable between sequence libraries of different sizes. Thus, this allows tackling the problem of taxonomic identification from a single big BLAST results by running several different BLASTs against several taxonomic subdivisions of a sequence library (e.g., NCBI’s NR, Swissprot, Uniprot [29]). However, this imposes to predefine a priori, candidate donor taxa and to run multiple BLASTs against different libraries that have either to be downloaded and formatted by the user or constructed by the user. The HGT index method was first used to determine genes of non-metazoan origin in the transcriptome of the bdelloid rotifer *Adineta ricciae* [28] and later used to infer the contribution of HGT to the transcriptomes of several different metazoan species, including vertebrates [11].

A refined scoring method has been recently proposed that not only takes into account sequence similarity between receiver and candidate donors, measured in BLAST bit score, but also taxonomic distance measured as the number of step in the NCBI’s taxonomic lineage to reach the common ancestor of the query and subject species [30]. The software is publicly available and has been initially developed to identify HGT in fungal genomes. However, this requires local installation of voluminous data, manual configuration, and expert skills are required to tune and adapt the method to identify HGT in other phyla.

Similarly, a method called DarkHorse and initially developed to identify HGT in bacteria is available as a software that can be downloaded and installed locally [31]. This method proposes a lineage probability index score (LPI) based on the taxonomic ranks of top hits to identify candidate HGT from BLAST against protein databases. Here again, the method can be generalized to other taxa of interest. However, the installation is restricted to users with computational skills on a Unix-based platform and requires installing, configuring and managing a MySQL database as well as downloading the whole NCBI’s NR database and the NCBI’s taxonomy.

Phylogenetic methods that compare a gene or protein tree to a reference species tree and identify inconsistencies are the gold standard to predict HGT. While such methods exist [32,33,34,35] they need to be implemented by expert users. These phylogeny-based methods require a reference species tree for comparison. However, such reference trees are not always available for the group of species of interest but some methods propose to generate a reference species trees as well as computing individual gene trees in parallel [36]. Furthermore, producing phylogenetic trees for a whole proteome or large gene set can be extremely time consuming, especially if the initial homology search retrieves numerous homologs for alignments and phylogenetic reconstruction. Because of their computationally demanding nature, phylogeny-based methods are currently hardly adapted to analysis of large genomes or to a high number of genes. Hence, AI or HGT-index based methods constitute an interesting method to rapidly identify candidate HGT and narrow down the number of genes to be phylogenetically analyzed afterwards.

As far as we know, there is currently no publicly available web tool that allows to easily and rapidly identify HGT from large datasets directly from a BLAST result.

Here, we propose Alienness, a user-friendly web application that requires no installation of any software and that is publicly accessible at http://alienness.sophia.inra.fr. Alienness requires nothing else than BLAST results against any sequence library at the NCBI and a few parameters to calculate AI for a set of query sequences (e.g., a whole proteome). Alienness can be applied to any genome of interest and to identify candidate HGT from any donor to any recipient taxonomic group.

We tested the accuracy of Alienness on the genomes of two plant-parasitic nematodes, for which phylogenetically supported HGT of a whole series of genes involved in plant parasitism had been previously identified [37,38]. We found that all phylogenetically supported cases could be retrieved by Alienness with an AI > 9 and that this AI threshold corresponded to a low rate of putative false positives.

We believe Alienness will promote a more rapid exploration of candidate HGT across the tree of life and will contribute to assessing more globally the evolutionary significance of HGT.

## 2. Materials and Methods

### 2.1. Input Data

**Figure 1:**
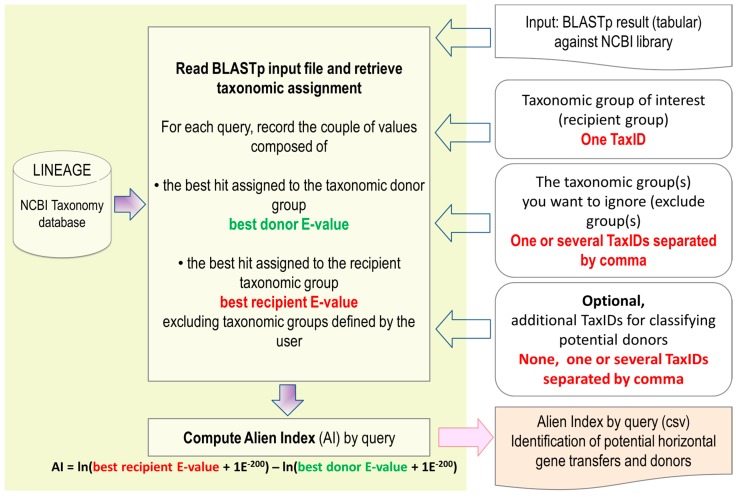
Schematic flow chart of Alienness processing BLAST results and providing Alien Indexes (AIs). NCBI: National Center for Biotechnology Information; TaxID: taxonomy identifier.

Typically, Alienness takes as input a compressed BLAST result (in .zip or .gz format) of a proteome performed against any NCBI protein database (Figure 1). Currently, the uploaded compressed BLAST result must not exceed a size of 500 Mb (ca. 2 to 2.5 Gb uncompressed). The BLAST result must be obtained by the BLASTp program available at the NCBI FTP website in BLAST+ package. The required format is the tabular format with comment lines obtained by using the -outfmt 7 BLAST option. The user must also define the NCBI taxonomy identifier (TaxID) of the taxonomic group of interest (recipient group, e.g., Metazoa, Viridiplantae) and one or several TaxIDs separated by a comma for the taxonomic groups to be ignored for the calculation of the AI (excluded group(s)). The latter parameter must at least include the TaxID of the query species used to produce the BLAST result to avoid self-BLAST results preventing the identification of putative HGT. Any hit to any taxon included in the entered taxonomic node(s) will be ignored and excluded from the calculation of AI. How taxonomic information is retrieved from BLAST tabular result is explained in the next section. By default, the different BLAST hits are categorized in the root nodes of the NCBI taxonomy: Archaea, Bacteria, Eukaryota, Viroids and Viruses. The groups ‘other’ and ‘unclassified’ are ignored, as they cannot be assigned to a known taxon. The user can define any additional category to classify the BLAST results in groups of interest (e.g., Fungi, Gammaproteobacteria, Deltavirus…). For this, the user just need to input a list of one or several corresponding TaxIDs.

### 2.2. Taxonomic Assignement

The taxonomic assignment of BLAST hit sequences is performed using the taxonomic databases available on the NCBI FTP site (ftp://ftp.ncbi.nlm.nih.gov/pub/taxonomy/). This repository contains the full taxonomy database along with flat files associating nucleotide and protein sequence records with their TaxIDs. The challenge lies in reconstructing the taxonomic lineage of species just from GenBank identifier (GI) extracted from BLAST results. To perform this taxonomic identification, we use the files ‘gi_taxid_prot.dmp.gz’ and ‘taxdump.tar.gz’, both available for download at ftp://ftp.ncbi.nlm.nih.gov/pub/taxonomy/. The first file provides, for each GI of protein record, its corresponding TaxID) The second file contains NCBI Taxonomy database dump files, and more precisely nodes.dmp and names.dmp files. Alienness retrieves the parent node ID of each node ID in GenBank taxonomy database from nodes.dmp file and associates a name for each of them using names.dmp file. Thus, Alienness re-builds the lineage of species based on the GI accessions.

### 2.3. Processing

The BLAST result of each query protein is read from the best BLAST hit to the last significant hit (Figure 1). Using the extracted taxonomic information (see above), Alienness records a couple of values composed of the best hit assigned to the taxonomic donor group called best donor E-value and the best hit assigned to the recipient taxonomic group called best recipient E-value. When a taxonomic assignment matches a group to be excluded, as defined by the user or belongs to the ‘Unclassified’ and ‘Other’ categories, the hit is ignored and discarded from the rest of the analyses.

### 2.4. Calculation of an Alien Index 

To illustrate the calculation of an AI we will take as an example identification of candidate HGT of non-metazoan origin in a metazoan genome. The principle remains the same for identification of inter-phylum HGT to the genome of any species of interest from any donor (alien) taxonomic groups.

Typically, Alienness takes as input the result of a BLASTp search of a whole set of predicted proteins of interest (e.g., from a whole genome or a transcriptome) against the NCBI’s NR library or any protein library available at NCBI. The BLAST result must be in tabular format and when performed against NR, we recommend using an E-value threshold of 1E^−3^ and no filtering for low complexity regions. Based on the BLAST hits GI accession numbers and on the NCBI taxonomy, Alienness extracts the best metazoan and best non-metazoan E-values to calculate an AI, first defined in [9], with the following formula.

$$AI=\;\ln\left(best\;metazoan\;Evalue+\;1E^{-200}\right)-\;\ln\left(best\;non\_metazoan\;Evalue+\;1E^{-200}\right)$$

When either no metazoan or non-metazoan significant BLAST hit is found, a penalty E-value of 1 is automatically assigned as the best metazoan or non-metazoan E-value, respectively. Hence, E-values of the best metazoan and non-metazoan hits vary between 0 and 1 and, consequently AI scores vary between −460.5 and 460.5. An AI > 0 indicates a better hit to a non-metazoan species than to a metazoan species and possible acquisition via HGT of non-metazoan origin. However, if the difference of magnitude between the best metazoan and non-metazoan E-values is low, this difference might not be significant and could just reflect a slight difference in the ranking of the best hits. For instance, a highly conserved protein between metazoan and non-metazoan species could by chance return a better hit to non-metazoan than metazoan. In the original publication of AI calculation, Gladyshev et al. [9] estimated, based on a few phylogenetic analyses that an AI ≥ 45 corresponds to HGT candidates highly supported by phylogenetic trees. They thus suggested that an AI threshold of 45 is a good indicator for likely HGT. In the results section of this paper, we re-evaluated this threshold on the genomes of plant-parasitic nematodes and showed that even lower AI scores provide a good balance in terms of sensitivity (recall of phylogenetically supported HGT) and specificity (low number of false positives).

## 3. Results

### 3.1. Alienness Web Portal and Interface

The Alienness web portal has been developed in HTML5 and CSS3 and launches a series of Perl programs via a CGI Perl module. Alienness is publicly and freely available at this URL: http://alienness.sophia.inra.fr and does not require creation of an account or downloading any software. Alienness just requires a pre-computed BLAST result in tabular format (-outfmt 7) against a protein library. The BLAST result file must be compressed in .gz or .zip format. The protein library must have GI or accession numbers that exist in the NCBI taxonomy database to retrieve the taxonomic information necessary to calculate AI values (i.e., any downloadable BLAST protein library at the NCBI). For a better coverage of the biodiversity, we recommend using the NCBI’s NR library. 

**Figure 2:**
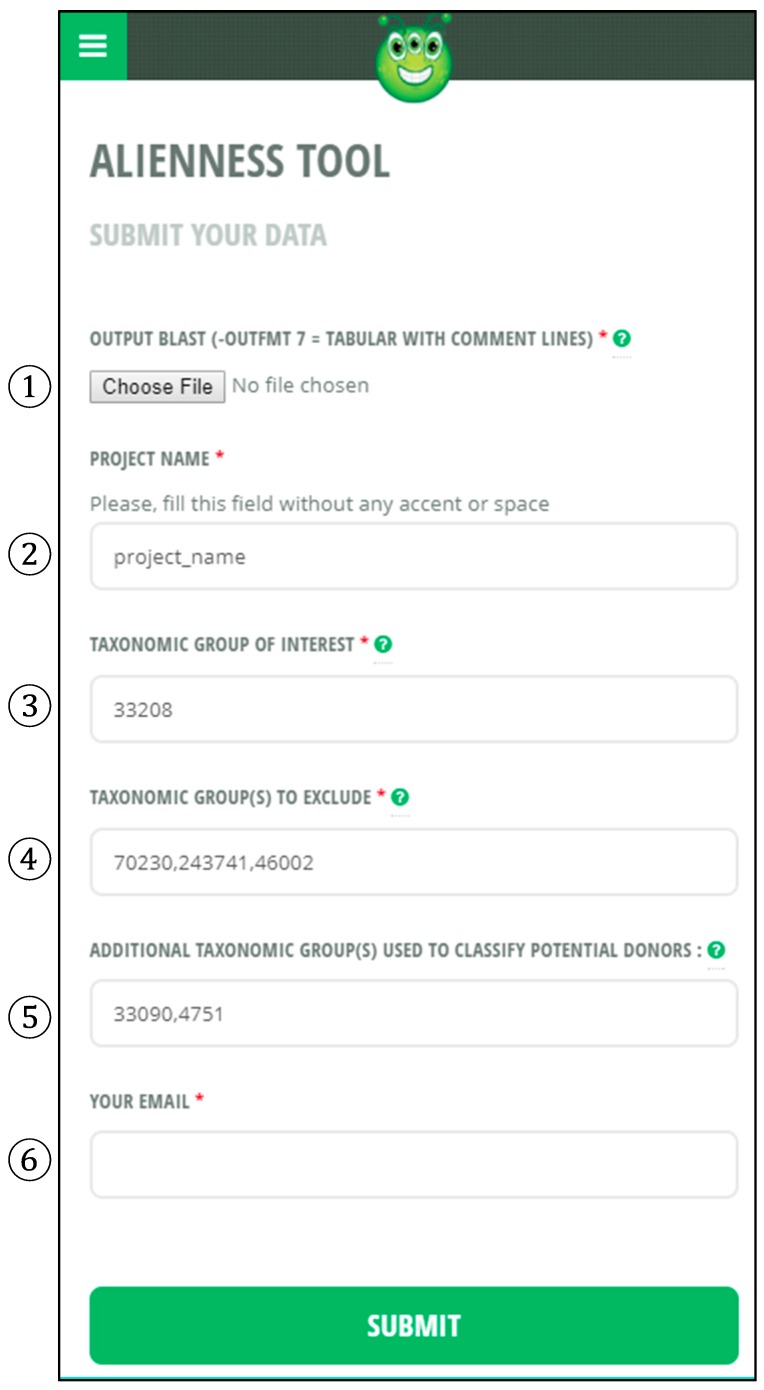
Graphical interface of Alienness to upload BLAST results and input the different parameters. Detailed information on 1–6 is given in the main text.

To run an Alienness analysis, click the ‘Alienness Tool’ tab and the following form will appear (Figure 2).

Fill the following information (all fields in Figure 2 except field 5 are mandatory).

1. Upload your BLAST result file here in .gz or .zip format.
2. Give a name to your project.
3. Enter the NCBI TaxID of the recipient group of interest (only 1 TaxID should be put here). For instance, if you are interested in HGT of non-metazoan origin to a metazoan species, please input 33208 (NCBI TaxID for Metazoa) as recipient. If you are interested in HGT of non-green plant origin to a green plant species, please input 33090 (NCBI TaxID for Viridiplantae). This is valid for any TaxID and this information is necessary to retrieve the best ‘TaxID of recipient’ E-value and best ‘TaxID of candidate donor’ E-value for calculation of an Alien index.
4. Enter the NCBI TaxIDs (one or several) for the taxonomic groups you want to ignore in the calculation of the AI. You must at least input the TaxID of the query species you used to produce the BLAST result. For instance, imagine that your query proteome is from *Mus musculus* and you are looking for HGT of non-metazoan origin. Most of the best BLAST hits (if not all) will be self-hits of *M. musculus* against itself. Hence, there is no chance to identify a *M. musculus* protein that would return a better hit to a non-metazoan than to a metazoan species. It is thus necessary to ignore all self-hits to *M. musculus* for the calculation of AI values. In this case, you have to input 10090 (NCBI TaxID for *M. musculus*). If you suspect that some HGT may have arisen earlier, in an ancestor of the rodents, for instance, then you have to input 9989 (TaxID for Rodentia). This will ignore all BLAST hits to Rodentia. Note that you can input several TaxIDs separated by comma and no space in this field if you want to ignore several non-overlapping taxonomic groups. This is useful if there is no monophyletic group in the NCBI taxonomy corresponding to the ensemble of species you want to ignore. Because Alienness is fast, you can also run it several times with different ignored TaxIDs and check the effect of this parameter on the number of candidate HGT identified (e.g., Rodentia, Mammalia, Vertebrata, etc.).
5. This field is optional and does not affect calculation of AI scores. This field controls the classification of the best BLAST hits in taxonomic categories in the output files. If left blank, the best hits are classified in the NCBI taxonomy basal categories Archaea, Bacteria, Eukaryota, Viroids and Viruses (the NCBI categories ‘Other’ and ‘Unclassified’ are ignored as they cannot be assigned to a species). If a user wants to further classify the best hits in other categories any additional TaxID can be entered. For instance, if you want to further categorize the Eukaryota into Fungi, Stramenopiles and Viridiplantae, you have to input 4751, 33634 and 33090 (separated by comma and no space).
6. Input your e-mail address. Please double check that your e-mail is correct because the link to download the results will be sent to this address.

Then click the submit button and wait for your compressed BLAST results to be uploaded to the server (do not refresh the page). Once the compressed BLAST result file is uploaded, a page will open and notify that the upload was successful and that you will receive two automatic e-mails. A first e-mail confirming the submission with a summary of the parameters selected by the user. A second one, a few minutes later, indicating that the job is finished and providing a summary of your job and a link to download a .zip archive with the results.

So far, we have run a dozen of eukaryotic whole proteomes with Alienness, and on average, the whole process from retrieval of taxonomic information to calculation of AI scores and production of result files takes 10–15 min (excluding upload time for the compressed BLAST results).

### 3.2. Results produced by Alienness

Once you download and uncompress the .zip result archive via the link in the automatic e-mail (Alienness_jobnumber.zip), the following result files will be available:

- Project_name_HGT_ai_full.csv: table presenting AI values and taxonomic information for all the proteins that returned an AI value.
- Project_name_HGT_ai_junk.csv: all the proteins that have an AI ≤ 0 and are thus probably not originated by HGT.
- Project_name_HGT_ai_likely_contamination.csv: proteins with an AI > 0 and ≥70% identity to a protein from candidate donors (these cases are considered as possible contaminations and should be carefully manually examined).
- Project_name_HGT_ai_likely_contamination_stat.csv: statistics on the taxonomic distribution (species and kingdoms) of candidate donors for the possible contamination category.
- Project_name_HGT_ai_possible.csv: proteins with an AI > 0 and <70% identity to candidate donors constitute the pool of possible HGT.
- Project_name_HGT_ai_possible_stat.csv: statistics on the taxonomic distribution (species and kingdoms) of candidate donors for the possible HGT category.
- Project_name_HGT_ai_very_likely.csv: proteins with an AI > 30 and <70% identity to candidate donors constitute the pool of very likely HGT.
- Project_name_HGT_ai_very_likely_stat.csv: statistics on the taxonomic distribution (species and kingdoms) of candidate donors for the very likely HGT category.
- Project_name_HGT_blastp_no_hits.csv: proteins for which no AI could be calculated because they returned no significant BLAST hits.
- Project_name_index.html: an html file that allows visually exploring the BLAST results with a color code.
- Project_name_summary.txt: log file providing information on execution time, parameters selected by the user, etc.

Note that the files Project_name_HGT_ai_full.csv, Project_name_HGT_ai_junk.csv, Project_name_HGT_ai_likely_contamination.csv, Project_name_HGT_ai_possible_stat.csv and Project_name_HGT_ai_very_likely.csv all have the same template (“Project_name” is substituted by the user-defined name set in the Alienness field number 2 in Figure 2). To exemplify this template, we use the .csv result file (very likely AI) of Alienness run on the 2008 version of the protein set of the root-knot nematode *Meloidogyne incognita* [39] (Supplementary Table S1).

- Column 1 (AI): the Alien Index
- Column 2 (query hits number): the number of hits returned by the protein in consideration.
- Column 3 (query name): the query accession number.
- Columns 4–8: the best E-values for the basal NCBI taxonomy categories Archaea, Bacteria, Eukaryota, Viroids and Viruses. When the best E-value does not belong to the taxonomic category in consideration, a “-” character is present.
- Columns 9–10 (optional): the best E-values for the user-defined additional taxonomic categories (field 5 of the form in Figure 2). In this example, there are two user-defined additional categories: Viridiplantae and Fungi.
- Column 11: the best E-value for the user-defined taxonomic group of interest (recipient group). In this example, the group of interest is Metazoa as we are interested in candidate HGT of non-metazoan origin in a metazoan species (the root-knot nematode *M. incognita*).
- Column 12 (best hit GI) *: the NCBI GI accession of the best BLAST hit.
- Column 13 (best hit prct ident) *: the percent identity between the query sequence and the best hit.
- Column 14 (best hit org nickname) *: an abbreviated species name for the best hit.
- Column 15 (best hit org full name) *: full species name of the best hit
- Column 16 (best hit taxo group) *: abbreviated taxonomic classification of the best hit.
- Column 17 (best hit taxid) *: NCBI TaxID of the best hit.
- Column 18 (best hit lineage): full taxonomic lineage for the best hit.

* Note that hits to the user-defined excluded taxonomic groups (field 4 of form in Figure 2) are ignored as well as hits to the NCBI categories ‘Other’ and ‘Unclassified’.

In addition, all the files named with a “_stat” suffix are built on a same two-column template and provide basic statistics on the candidate donors (or contaminant). The first column contains names of kingdom or taxa and the second the occurrence of these taxa in the set of candidate donors or possible contaminants.

**Figure 3:**
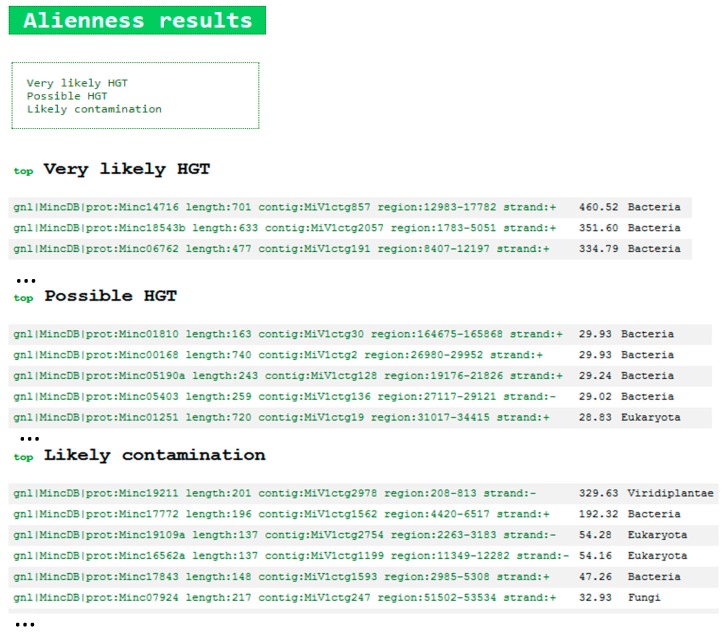
The .html index file opened in a web browser to explore the BLAST results by category (Very likely HGT, Possible HGT and Likely contamination).

The file “Project_name_index.html” allows exploring the BLAST results of interest regarding candidate HGT and candidate contaminants. By opening this file in a web browser, the following page will open (Figure 3).

**Figure 4:**
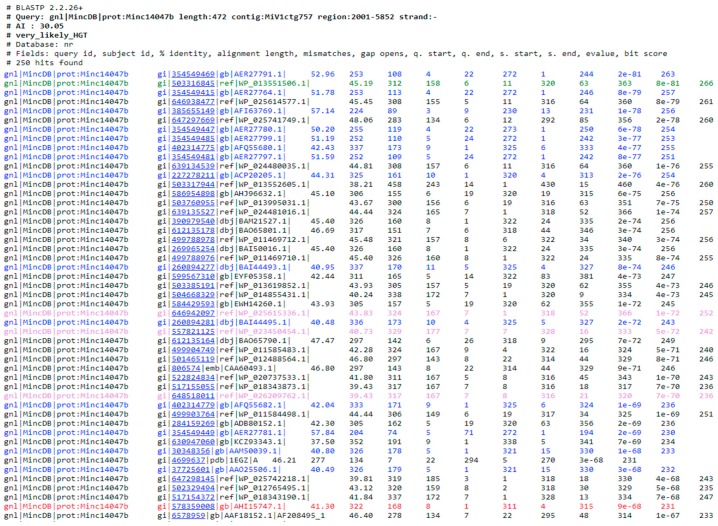
The color-coded BLAST results that can be explored from a web browser, color code and further explanations are detailed below.

BLAST results are classified in 3 categories, (i) very likely HGT (AI > 30 and <70% identity to candidate donor); (ii) possible HGT (AI > 0 and <70% identity to candidate donor) and (iii) likely contamination (AI > 0 and ≥70% identity to candidate donor). For each category, a list of accession numbers from the query proteome as well as the AI value and the taxonomic category of the candidate donor are indicated. By clicking on any of these accession numbers, a color-coded BLAST result in .html format opens and allows exploring the results in more details. An example on a *M. incognita* protein (Minc14047b, a GH5 cellulase acquired by HGT [37]) is given below (Figure 4).

In the BLAST result, the color code is as follows: hits that belong to the taxonomic group(s) to exclude are highlighted in blue. In this example, we chose to exclude the Tylenchida (TaxID: 6300) that include the root-knot nematodes and many other related plant-parasitic nematodes. The best ‘alien’ hit is indicated in green. In this example, we were interested in HGT of non-metazoan origin in a metazoan, so the alien taxon is anything but Metazoa. Here, the best alien taxon is from the bacterium *Cellulophaga algicola* with an E-value of 8E^−81^ The best hit that belongs to the taxonomic group of interest (=recipient taxon, as defined in the field 3 of the form in Figure 2) is highlighted in red. In this example, the taxonomic group of interest is Metazoa and the best metazoan hit is from the phytophagous insect *Apriona japonica* with an E-value of 9E^−68^. These are thus the green (here best non-metazoan) and red (here best metazoan) E-values that are used to calculate the AI. Hits for which a taxonomic group could not be assigned are highlighted in light violet. This corresponds to protein that have either been removed from the NCBI or replaced by another protein with a different accession number. Hits that belong to the categories ‘other’ and ‘unclassified’ are highlighted in light grey. Note that no color code is given after the best green and best red taxonomic groups have been identified because these are necessary and sufficient to calculate an AI.

### 3.3. Validation of Alienness on Nematode Genomes

**Figure 5:**
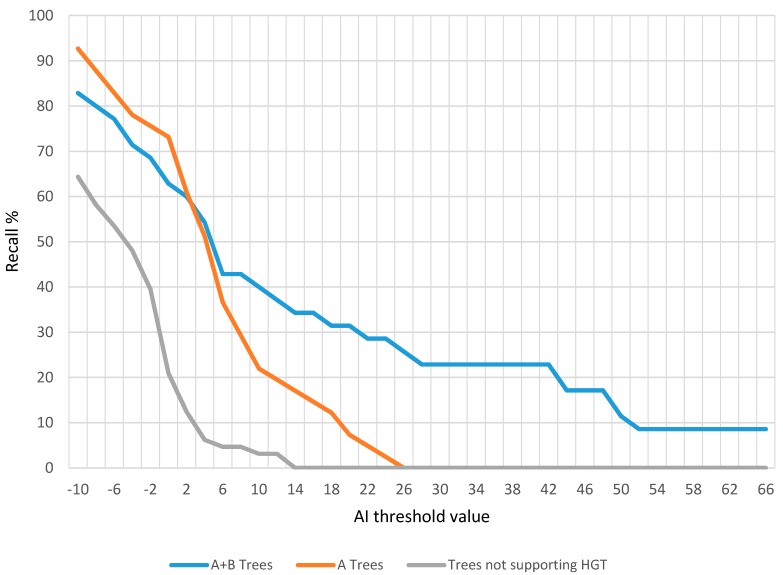
Recall rate (sensitivity) and risks of false positives of Alienness at different AI thresholds. The percentage of retrieved phylogenetically supported (with A or A + B nodes) as well as of phylogenetically unsupported cases (*y*-axis) as a function of the AI threshold (*x*-axis) is represented on this graph.

Alienness identified 649 proteins with an AI > 0 (i.e., that have better hits to non-metazoan species than to metazoan) on the 2008 version of the *M. incognita* proteome [39]. Some of these proteins could originate from HGT events. To assess which AI threshold maximizes the number of very likely HGT and minimizes the number of false positives, we compared the results of Alienness to a previous analysis we published on the same proteome in 2012 [40]. Briefly, in this 2012 analysis we first identified *Meloidogyne* proteins having no clear ortholog in Metazoa via an OrthoMCL analysis [41]. We used these proteins to reconstruct automatic phylogenies with FIGENIX [32] and then searched for topologies supporting an HGT event using PhyloPattern [33]. We identified 205 *M. incognita* proteins for which an AI value could be calculated by Alienness and an automatic phylogenetic tree was previously reconstructed (Supplementary Table S2). To estimate the recall rate of Alienness, we plotted the percentage of candidate HGT proteins supported by phylogenies (Figure 5) for different AI threshold values (from −10 to >66 with incremental steps of 2). We differentiate phylogenies only supported by a node ‘A’ linking the plant-parasitic nematodes to non-metazoan putative donors and those supported by both a node ‘A’ and an additional node ‘B’, linking the node ‘A’ to any species but plant-parasitic nematodes [40]. To estimate the risk of false positives produced by Alienness, we plotted the percentage of proteins returning phylogenetic trees not supporting HGT at the same range of AI values.

At an AI value > −10 we recall 83% and 93% of HGT cases supported by automatic phylogenies with A + B and A support, respectively. However, at this AI value, 64% of the proteins that returned phylogenies without support for HGT are also retrieved. Note that the AI cut-off as to be set to >−37 to retrieve all phylogenetically supported proteins, but at this threshold, 93% of the proteins returning trees without support for HGT are also retrieved. At an E-value > 0; 63% of the HGT proteins with A + B phylogenetic support are retrieved and 68% of those with A support only. However, at this threshold, 21% of the proteins returning trees without support for HGT are also retrieved. From an AI threshold of >14, all proteins returning trees not supporting HGT are eliminated. An AI > 14 corresponds to a difference of magnitude >1E^6^ between the best non-metazoan and best metazoan hits. At this threshold, 34% and 17% of the A + B and A trees, respectively are still retrieved. At an AI threshold >26, only A + B phylogenetically supported proteins remain, the A-only supported cases are eliminated.

To further measure the ability of Alienness to recall highly supported candidate HGT, we tested whether previously reported cases of HGT, supported by meticulous manual phylogenetic analysis were found by Alienness. From a survey of the literature, we identified 23 cases of HGT in plant-parasitic nematodes supported by manual phylogenetic analysis (Table 1, Supplementary Table S3).

We retrieved the PFAM domains [67] present in the corresponding proteins and scanned these domains against the proteomes of the root-knot nematode *M. incognita* [39] and of the cyst nematode *G. rostochiensis* [19]. We found corresponding protein-coding genes for 13 and 19 of these HGT cases in the proteomes of *M. incognita* and *G. rostochiensis*, respectively. We reported the AI scores for the proteins corresponding to these HGT cases (Table 1, Supplementary Table S3). In some of the cases, the genes acquired via HGT form multigene families in the recipient species genomes [37]. In these cases, we reported the highest AI value and indicated how many more proteins were from this gene family in Supplementary Table S3.

**Figure 6:**
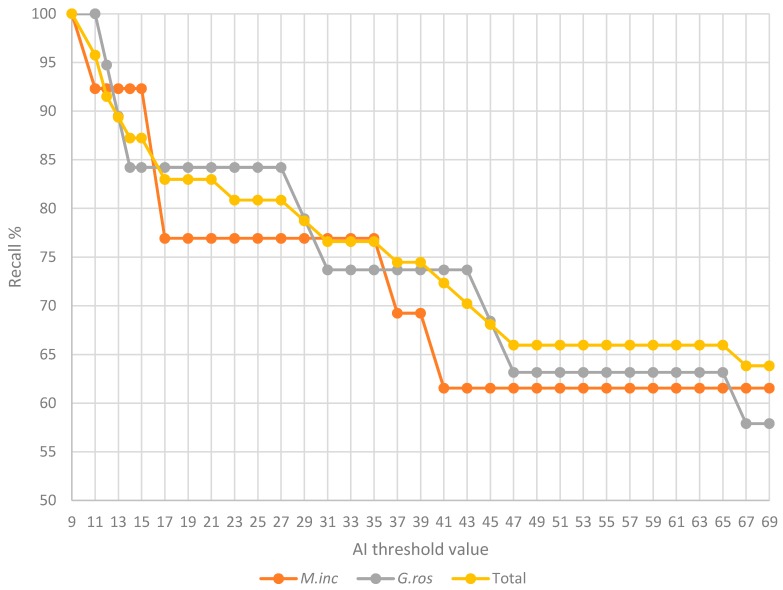
Phylogenetically supported HGT from the literature in the genomes of two plant-parasitic nematodes at different AI thresholds. Percentage of known and phylogenetically supported HGT cases (*y*-axis) at different AI thresholds (*x*-axis) in *M. incognita* (orange) and *G. rostochiensis* (grey).

We found that 100% of the phylogenetically supported cases returned an AI > 9 in *M. incognita* and an AI > 11 in *G. rostochiensis* (Figure 6). With an AI value > 14; more than 92% and 84% of the phylogenetically supported cases are retrieved in *M. incognita* and *G. rostochiensis*, respectively. As mentioned in the previous section, at this AI threshold, no case of automatic phylogeny not supporting HGT is retrieved. 

Overall, depending on the aim of the user, we recommend using different AI thresholds. To have a more comprehensive overview of possible HGT, we recommend an AI > 0 but with a substantial proportion of possible false positives, to be manually curated. To focus on candidates that are likely to produce phylogenetic trees supporting HGT, and minimizing the rate of false positives, we recommend an AI > 14. To focus on the candidates that will likely produce phylogenies with higher support for HGT, we recommend an AI > 26.

## 4. Discussion

Alienness allows rapid identification of candidate HGT from whole proteomes and provides accompanying taxonomic information as well as basic statistics for the possible donors. An advantage of providing scores such as an AI is that the user can define its own threshold of significance and partition its dataset according to the AI scores. A high AI is an indication for putative acquisition by HGT. Obviously; a careful phylogenetic analysis remains the gold standard to support the hypothesis of HGT. However, careful phylogenetic analysis is time-consuming, can be complicated and requires expert skills, which is not (or hardly) applicable to large datasets of thousands of genes. In this perspective, Alienness provides an efficient metrics to quickly reduce the number of candidates that can be further analyzed via phylogenetic analysis. For instance, in the case of the plant-parasitic nematodes 20,359 and 14,309 proteins were present in the predicted proteome of *M. incognita* and *G. rostochiensis*, respectively. However, only 632 *M. incognita* and 519 *G. rostochiensis* proteins returned an AI > 0 and <70% identity to putative donors, which substantially narrows down the number of phylogenetic analyzes that must be undertaken. With an AI > 14, corresponding to low risk of false positives, the number of candidates is reduced to 165 in *M. incognita* and 136 in *G. rostochiensis*.

Alienness also provides a list of putative contaminants that can be useful for the user to prune the original dataset or even to identify possible endosymbiont. Any protein with an AI > 0 and ≥70% identity is put apart in a category of possible contaminants. In a recent paper, Ku and Martin [68] defined what they called the 70% rule, above which candidate HGT of prokaryote origin to eukaryotes are more likely to represent contamination than actual HGT. This rule holds at the nucleotide level and for prokaryote to eukaryote transfers. In our own experience, none of the candidate HGT of non-metazoan origin to nematodes, supported by careful phylogenetic analysis, reported so far present more than 70% identity at the protein level with a candidate donor. We thus, extended this 70% rule to the protein level. However, among these suspicious contaminants, rare recent or well-conserved HGT might be present, but we recommend being particularly cautious with these candidates to rule out the possibility of contamination. One evident first verification is to check that bona-fide genes from the recipient species surround candidate HGT genes in its genome. With Alienness, this can be easily achieved by looking for surrounding genes with negative AI values. A series of additional checks hold true for prokaryotic to metazoan HGTs, including presence of spliceosomal introns on the gene models, or presence of eukaryotic signal peptides for secretion in the protein. These and other features specific to the recipient species and absent or much less frequent in the candidate donors are good indicators to rule out the hypothesis of contamination. Support from expression data is also an independent element making HGT more likely than contamination.

Although Alienness provides rapid identification of candidate HGT, some limitations should be kept in mind while interpreting the results. First, as discussed in the previous section, Alienness just predicts candidate HGT that ideally need to be further supported by additional sourced of evidence. Second, the ability to detect candidate HGT depends on the quality and coverage of the queried sequence library. Both the accuracy of the taxonomic assignment and the diversity of taxa covered by the reference library influence the calculation of AI or any other method based on BLAST hits. Third, because BLAST E-values and bit scores also depend on the length of the query protein and matching hits, metrics such as AI and HGT Index will necessarily return lower scores for short sequences as the difference of E-value or bit score between the best donor and recipient organisms will have less difference in magnitude.

Overall, Alienness provides a fast and reliable service to rapidly detect candidate HGT in any genome of interest from any donor group. Although HGT is recognized as an evolutionary important and prevalent mechanism in prokaryotes, its importance and prevalence in eukaryotes remain actively debated and can be controversial [69]. Exploring more proteomes for a wider diversity of species will allow deciphering the contribution of HGT to the biology and genomes across the tree of life more comprehensively. Alienness will promote this effort by publicly offering a user-friendly method to rapidly scan existing and upcoming genomes.

## Figures and Tables

**Table 1:** Horizontal gene transfer (HGT) cases supported by manual phylogenetic analysis in root-knot and cyst nematodes.

Protein / Protein Family [refs]	Highest AI in *M. incognita*	Highest AI in *G. rostochiensis*	Process
GH5_2 Cellulases [37,42,43,44,45,46,47]	39.14	198.94	PCW Degradation
GH30 Xylanase [37,48]	259.49	-	PCW Degradation
GH28 Polygalacturonase [37,49]	351.60	-	PCW Degradation
Expansin-like proteins [37,39,50,51]	86.11	29.93	PCW Degradation
GH43 candidate Arabinanase [37] *	69.07	-	PCW Degradation
GH53 candidate Arabinogalactanase [52] *	-	349.30	PCW Degradation
PL3 Pecate Lyase [37,53,54,55]	137.46	137.06	PCW Degradation
Chorismate Mutase [56,57,58]	15.02	42.36	Def. manipulation
Candidate Isochorismatase [59] *	91.41	66.08	Def. manipulation
Candidate Cyanate Lyases [60,61] *	9.90	11.51	Detoxification
GH32 invertase [62]	154.42	241.26	Nutrient processing
VB1 thiD [63] *	-	154.50	Nutrient processing
VB1 thiE [63] *	-	163.99	Nutrient processing
VB1 thi4 [63] *	-	108.07	Nutrient processing
VB1 thiM [63] *	-	46.05	Nutrient processing
VB1 tenA [63] *	-	108.33	Nutrient processing
VB5 panC [63] *	16.52	183.11	Nutrient processing
VB6 SNO [64]	-	-	Nutrient processing
VB6 SOR-SNZ [64]	-	12.72	Nutrient processing
Candidate GSI Glutamine Synthase [40,65] *	35.59	29.24	Nutrient processing
NodL—like [65,66] *	-	13.12	Feed. site induction
Candidate L-threonine aldolase [40,65] *	-	164.69	??
Candidate Phosphorybosyl transferase [40,65] *	202.63	198.13	??

* function not biochemically confirmed so far; PCW: Plant Cell Wall; AI: Alien Index; ??: no inferred process

## Supplementary Materials

The following are available online at www.mdpi.com/2073-4425/8/10/248/s1. Table S1: AI-MiV1: Alien Indexes of the *M. incognita* whole proteome, Table S2: AI-Tree-MiV1: Alien Indexes of HGT supported by automatic phylogeny, Table S3: GeneHGT: Alien Indexes of previously reported HGT in plant-parasitic nematodes.
