# Supplementary material for: Alienness: Rapid Detection of Candidate Horizontal Gene Transfers across the Tree of Life
Source: Genes (Basel). 2017 Sep 29;8(10):248. doi: 10.3390/genes8100248 (PMC5664098; doi:10.3390/genes8100248)
Supplement: Supplementary file 1 [file genes-08-00248-s001.zip › Supp-TableS3-GeneHGT_edit.docx]

| **Supplementary Table S3.** GeneHGT: Alien Indexes of previously reported HGT in plant-parasitic nematodes. | | | | | | | | |
| --- | --- | --- | --- | --- | --- | --- | --- | --- |
| **Gene / gene family** | **Pfam domain** | **From ref.** | **Highest AI in MiV1** | **MiV1 accession** | **Highest AI in G.ros** | **G. ros accessions** | **Function** | **Process** |
| GH5_2 Cellulases | PF00150 Cellulase (glycosyl hydrolase family 5) | [1–7] | 39.14 | Minc12674 (+22 other) | 198.94 | GROS_g11949  (+10 other) | Cellulose degradation | Plant Cell Wall Degradation |
| GH30 xylanase | PF02055  Glycosyl hydrolase family 30 TIM-barrel domain | [7,8] | 259.49 | Minc11164 (+5 other) | - | - | Xylan degradation | Plant Cell Wall Degradation |
| GH28 Polygalacturonase | PF00295  Glyco_hydro_28 | [7,9] | 351.60 | Minc18543b (+3 other) | - | - | Pectin decorations degradation | Plant Cell Wall Degradation |
| Expansin-like proteins | PF03330  Rare lipoprotein A (RlpA)-like double-psi beta-barrel | [7,10–12] | 86.11 | Minc07960 (+7 other) | 29.93 | GROS_g11727  (+6 other) | Softening of non-covalent bonds | Plant Cell Wall Degradation |
| GH43 candidate Arabinanase | PF04616  Glyco_hydro_43 | [7]* | 69.07 | Minc10639 (+1 other) | - | - | Pectin decorations degradation | Plant Cell Wall Degradation |
| GH53 candidate Arabinogalactan endo-1,4-beta-galactosidase | PF07745  Glycosyl hydrolase family 53 | [13]* | - | Specific to Globodera (so far) | 349.30 | GROS_g08150 | Pectinose / arabinogalactan degradation | Plant Cell Wall Degradation |
| PL3 Pecate Lyase | PF03211  Pectate lyase | [7,14–16] | 137.46 | Minc05972 (+30 other) | 137.06 | GROS_g04366  (+2 other) | Pectin degradation | Plant Cell Wall Degradation |
| GH32 invertase | PF00251  Glycosyl hydrolases family 32 N-terminal domain | [17] | 154.42 | Minc09960 | 241.26 | GROS_g08674  (+10 other) | Degradation of sucrose in glucose + fructose | Nutrient processing |
| Chorismate Mutase | PF01817  Chorismate mutase type II | [18–20] | 15.02 | Minc07025 | 42.36 | GROS_g08190  (+1 other) | Conversion of Chorismate into SA | Plant defense manipulation |
| Candidate Isochorismatase | PF00857  Isochorismatase family | [21]* | 91.41 | Minc14448 | 66.08 | GROS_g01640 | Conversion of Chorismate into SA | Plant defense manipulation |
| Candidate Cyanate Lyases | PF02560  Cyanate lyase C-terminal domain | [22,23] * | 9.90 | Minc06015 (+1 other) | 11.51 | GROS_g09531 |  | Detoxification |
| VB1 thiD | PF08543  Phosphomethylpyrimidine kinase | [24]* | - | There are proteins containing a PF08543 domain but none with an AI>0 | 154.50 | GROS_g07352 | Vitamin B1 biosynthesis | Nutrient processing |
| VB1 thiE | PF02581  Thiamine monophosphate synthase/TENI | [24]* | - | Specific to globodera (so far?) | 163.99 | GROS_g07353 | Vitamin B1 biosynthesis | Nutrient processing |
| VB1 thi4 | PF01946  Thi4 family | [24]* | - | - | 108.07 | GROS_g10855 | Vitamin B1 biosynthesis | Nutrient processing |
| VB1 thiM | PF02110  Hydroxyethylthiazole kinase family | [24]* | - | - | 46.05 | GROS_g07354 | Vitamin B1 salvage | Nutrient processing |
| VB1 tenA | PF03070  TENA/THI-4/PQQC family | [24]* | - | - | 108.33 | GROS_g05327  GROS_g07355 | Vitamin B1 salvage | Nutrient processing |
| VB5 panC | PF02569  Pantoate-beta-alanine ligase | [24]* | 16.52 | Minc14603 | 183.11 | GROS_g05752 | Vitamin B5 biosynthesis | Nutrient processing |
| VB6 SNO | PF01174  SNO glutamine amidotransferase family | [25] | - | - | - | GROS_g08955Ŧ | Vitamin B6 biosynthesi | Nutrient processing |
| VB6 SOR-SNZ | PF01680  SOR/SNZ family | [25] | - | - | 12.72 | GROS_g08956 | Vitamin B6 biosynthesis | Nutrient processing |
| NodL - like | PF12464  Maltose acetyltransferase  +  PF00132  Bacterial transferase hexapeptide (six repeats) | [26,27]* | Not in MiV1 |  | 13.12 | GROS_g11033 | Candidate acetyltransferase | Feeding site induction |
| Candidate GSI Glutamine Synthase | PF00120  Glutamine synthetase, catalytic domain | [27,28]* | 35.59 | Minc08077 (+3 other) | 29.24 | GROS_g02362 | Nitrogen assimilation | Nutrient processing |
| Candidate L-threonine aldolase | PF01212  Beta-eliminating lyase | [27,28]* | Not in MiV1 | Minc3s06087g39310  Minc3s03983g35231  Minc3s09176g42992 | 164.69 | GROS_g10423  GROS_g10421  GROS_g10422 | ?? | ?? |
| Candidate Phosphorybosyl transferase | PF00156  Phosphoribosyl transferase domain | [27][28]* | 202.63 | Minc16723 | 198.13 | GROS_g06735  GROS_g04632 | ?? | ?? |

* no functional characterization so far; Ŧ protein with an annotation problem in the genome, only the N-terminal part corresponds to SNO

1. Smant, G.; Stokkermans, J. P.; Yan, Y.; de Boer, J. M.; Baum, T. J.; Wang, X.; Hussey, R. S.; Gommers, F. J.; Henrissat, B.; Davis, E. L.; Helder, J.; Schots, A.; Bakker, J. Endogenous cellulases in animals: isolation of beta-1, 4-endoglucanase genes from two species of plant-parasitic cyst nematodes. *Proc Natl Acad Sci U A* **1998**, *95*, 4906–11.

2. Rosso, M. N.; Favery, B.; Piotte, C.; Arthaud, L.; De Boer, J. M.; Hussey, R. S.; Bakker, J.; Baum, T. J.; Abad, P. Isolation of a cDNA encoding a beta-1,4-endoglucanase in the root-knot nematode Meloidogyne incognita and expression analysis during plant parasitism. *Mol Plant Microbe Interact* **1999**, *12*, 585–91.

3. Bera-Maillet, C.; Arthaud, L.; Abad, P.; Rosso, M. N. Biochemical characterization of MI-ENG1, a family 5 endoglucanase secreted by the root-knot nematode Meloidogyne incognita. *Eur J Biochem* **2000**, *267*, 3255–63.

4. Yan, Y.; Smant, G.; Stokkermans, J.; Qin, L.; Helder, J.; Baum, T.; Schots, A.; Davis, E. Genomic organization of four β-1,4-endoglucanase genes in plant-parasitic cyst nematodes and its evolutionary implications. *Gene* **1998**, *220*, 61–70, doi:10.1016/S0378-1119(98)00413-2.

5. Davis, E. L.; Hussey, R. S.; Baum, T. J.; Bakker, J.; Schots, and A.; Rosso, M.-N.; Abad, and P. Nematode Parasitism Genes. *Annu. Rev. Phytopathol.* **2000**, *38*, 365–396, doi:10.1146/annurev.phyto.38.1.365.

6. Ledger, T. N.; Jaubert, S.; Bosselut, N.; Abad, P.; Rosso, M. N. Characterization of a new beta-1,4-endoglucanase gene from the root-knot nematode Meloidogyne incognita and evolutionary scheme for phytonematode family 5 glycosyl hydrolases. *Gene* **2006**, *382*, 121–8.

7. Danchin, E. G.; Rosso, M. N.; Vieira, P.; de Almeida-Engler, J.; Coutinho, P. M.; Henrissat, B.; Abad, P. Multiple lateral gene transfers and duplications have promoted plant parasitism ability in nematodes. *Proc Natl Acad Sci U A* **2010**, *107*, 17651–6.

8. Mitreva-Dautova, M.; Roze, E.; Overmars, H.; de Graaff, L.; Schots, A.; Helder, J.; Goverse, A.; Bakker, J.; Smant, G. A symbiont-independent endo-1,4-beta-xylanase from the plant-parasitic nematode Meloidogyne incognita. *Mol Plant Microbe Interact* **2006**, *19*, 521–9.

9. Jaubert, S.; Laffaire, J.-B.; Abad, P.; Rosso, M.-N. A polygalacturonase of animal origin isolated from the root-knot nematode Meloidogyne incognita. *FEBS Lett.* **2002**, *522*, 109–112, doi:10.1016/S0014-5793(02)02906-X.

10. Qin, L.; Kudla, U.; Roze, E. H.; Goverse, A.; Popeijus, H.; Nieuwland, J.; Overmars, H.; Jones, J. T.; Schots, A.; Smant, G.; Bakker, J.; Helder, J. Plant degradation: a nematode expansin acting on plants. *Nature* **2004**, *427*, 30.

11. Kudla, U.; Qin, L.; Milac, A.; Kielak, A.; Maissen, C.; Overmars, H.; Popeijus, H.; Roze, E.; Petrescu, A.; Smant, G.; Bakker, J.; Helder, J. Origin, distribution and 3D-modeling of Gr-EXPB1, an expansin from the potato cyst nematode Globodera rostochiensis. *FEBS Lett* **2005**, *579*, 2451–7.

12. Abad, P.; Gouzy, J.; Aury, J.-M.; Castagnone-Sereno, P.; Danchin, E. G. J.; Deleury, E.; Perfus-Barbeoch, L.; Anthouard, V.; Artiguenave, F.; Blok, V. C.; Caillaud, M.-C.; Coutinho, P. M.; Dasilva, C.; Luca, F. D.; Deau, F.; Esquibet, M.; Flutre, T.; Goldstone, J. V.; Hamamouch, N.; Hewezi, T.; Jaillon, O.; Jubin, C.; Leonetti, P.; Magliano, M.; Maier, T. R.; Markov, G. V.; McVeigh, P.; Pesole, G.; Poulain, J.; Robinson-Rechavi, M.; Sallet, E.; Ségurens, B.; Steinbach, D.; Tytgat, T.; Ugarte, E.; Ghelder, C. van; Veronico, P.; Baum, T. J.; Blaxter, M.; Bleve-Zacheo, T.; Davis, E. L.; Ewbank, J. J.; Favery, B.; Grenier, E.; Henrissat, B.; Jones, J. T.; Laudet, V.; Maule, A. G.; Quesneville, H.; Rosso, M.-N.; Schiex, T.; Smant, G.; Weissenbach, J.; Wincker, P. Genome sequence of the metazoan plant-parasitic nematode Meloidogyne incognita. *Nat. Biotechnol.* **2008**, *26*, 909–915, doi:10.1038/nbt.1482.

13. Vanholme, B.; Haegeman, A.; Jacob, J.; Cannoot, B.; Gheysen, G. Arabinogalactan endo-1,4-beta-galactosidase: a putative plant cell wall-degrading enzyme of plant-parasitic nematodes. *Nematology* **2009**, *11*, 739–747, doi:10.1163/156854109x404599.

14. Popeijus, H.; Overmars, H.; Jones, J.; Blok, V.; Goverse, A.; Helder, J.; Schots, A.; Bakker, J.; Smant, G. Degradation of plant cell walls by a nematode. *Nature* **2000**, *406*, 36–7.

15. Doyle, E. A.; Lambert, K. N. Cloning and Characterization of an Esophageal-Gland-Specific Pectate Lyase from the Root-Knot Nematode Meloidogyne javanica. *Mol. Plant. Microbe Interact.* **2002**, *15*, 549–556, doi:10.1094/MPMI.2002.15.6.549.

16. Kudla, U.; Milac, A.-L.; Qin, L.; Overmars, H.; Roze, E.; Holterman, M.; Petrescu, A.-J.; Goverse, A.; Bakker, J.; Helder, J.; Smant, G. Structural and functional characterization of a novel, host penetration-related pectate lyase from the potato cyst nematode Globodera rostochiensis. *Mol. Plant Pathol.* **2007**, *8*, 293–305, doi:10.1111/j.1364-3703.2007.00394.x.

17. Danchin, E. G. J.; Guzeeva, E. A.; Mantelin, S.; Berepiki, A.; Jones, J. T. Horizontal Gene Transfer from Bacteria Has Enabled the Plant-Parasitic Nematode Globodera pallida to Feed on Host-Derived Sucrose. *Mol. Biol. Evol.* **2016**, *33*, 1571–1579, doi:10.1093/molbev/msw041.

18. Lambert, K. N.; Allen, K. D.; Sussex, I. M. Cloning and Characterization of an Esophageal-Gland-Specific Chorismate Mutase from the Phytoparasitic Nematode Meloidogyne javanica. *Mol. Plant. Microbe Interact.* **1999**, *12*, 328–336, doi:10.1094/MPMI.1999.12.4.328.

19. Jones, J. T.; Furlanetto, C.; Bakker, E.; Banks, B.; Blok, V.; Chen, Q.; Phillips, M.; Prior, A. Characterization of a chorismate mutase from the potato cyst nematode Globodera pallida. *Mol. Plant Pathol.* **2003**, *4*, 43–50.

20. Vanholme, B.; Kast, P.; Haegeman, A.; Jacob, J.; Grunewald, W.; Gheysen, G. Structural and functional investigation of a secreted chorismate mutase from the plant-parasitic nematode Heterodera schachtii in the context of related enzymes from diverse origins. *Mol. Plant Pathol.* **2009**, *10*, 189–200, doi:10.1111/j.1364-3703.2008.00521.x.

21. Bauters, L.; Haegeman, A.; Kyndt, T.; Gheysen, G. Analysis of the transcriptome of Hirschmanniella oryzae to explore potential survival strategies and host–nematode interactions. *Mol. Plant Pathol.* **2014**, *15*, 352–363, doi:10.1111/mpp.12098.

22. Opperman, C. H.; Bird, D. M.; Williamson, V. M.; Rokhsar, D. S.; Burke, M.; Cohn, J.; Cromer, J.; Diener, S.; Gajan, J.; Graham, S.; Houfek, T. D.; Liu, Q.; Mitros, T.; Schaff, J.; Schaffer, R.; Scholl, E.; Sosinski, B. R.; Thomas, V. P.; Windham, E. Sequence and genetic map of Meloidogyne hapla: A compact nematode genome for plant parasitism. *Proc Natl Acad Sci U A* **2008**, *105*, 14802–7.

23. Wybouw, N.; Balabanidou, V.; Ballhorn, D. J.; Dermauw, W.; Grbić, M.; Vontas, J.; Van Leeuwen, T. A horizontally transferred cyanase gene in the spider mite Tetranychus urticae is involved in cyanate metabolism and is differentially expressed upon host plant change. *Insect Biochem. Mol. Biol.* **2012**, *42*, 881–889, doi:10.1016/j.ibmb.2012.08.002.

24. Craig, J. P.; Bekal, S.; Niblack, T.; Domier, L.; Lambert, K. N. Evidence for Horizontally Transferred Genes Involved in the Biosynthesis of Vitamin B-1, B-5, and B-7 in Heterodera glycines. *J. Nematol.* **2009**, *41*, 281–290.

25. Craig, J. P.; Bekal, S.; Hudson, M.; Domier, L.; Niblack, T.; Lambert, K. N. Analysis of a Horizontally Transferred Pathway Involved in Vitamin B6 Biosynthesis from the Soybean Cyst Nematode Heterodera glycines. *Mol. Biol. Evol.* **2008**, *25*, 2085–2098, doi:10.1093/molbev/msn141.

26. McCarter, J. P.; Mitreva, M. D.; Martin, J.; Dante, M.; Wylie, T.; Rao, U.; Pape, D.; Bowers, Y.; Theising, B.; Murphy, C. V.; Kloek, A. P.; Chiapelli, B. J.; Clifton, S. W.; Bird, D. M.; Waterston, R. H. Analysis and functional classification of transcripts from the nematode Meloidogyne incognita. *Genome Biol.* **2003**, *4*, R26, doi:10.1186/gb-2003-4-4-r26.

27. Scholl, E. H.; Thorne, J. L.; McCarter, J. P.; Bird, D. M. Horizontally transferred genes in plant-parasitic nematodes: a high-throughput genomic approach. *Genome Biol* **2003**, *4*, R39.

28. Paganini, J.; Campan-Fournier, A.; Da Rocha, M.; Gouret, P.; Pontarotti, P.; Wajnberg, E.; Abad, P.; Danchin, E. G. J. Contribution of Lateral Gene Transfers to the Genome Composition and Parasitic Ability of Root-Knot Nematodes. *PLoS ONE* **2012**, *7*, e50875, doi:10.1371/journal.pone.0050875.
